# The Role of Serum Uric Acid and Serum Creatinine Ratio as Possible Markers of Autonomic Dysfunction and Left Ventricular Mass Index in Atherosclerotic Renal Artery Stenosis

**DOI:** 10.3390/jcdd12060202

**Published:** 2025-05-28

**Authors:** Antonietta Gigante, Rosa Cascone, Chiara Pellicano, Francesco Iannazzo, Francesca Romana Gadaleta, Edoardo Rosato, Rosario Cianci

**Affiliations:** Department of Translational and Precision Medicine, Sapienza University of Rome, 00185 Rome, Italy; antonietta.gigante@uniroma1.it (A.G.); rosa.cascone@uniroma1.it (R.C.); chiara.pellicano@gmail.com (C.P.); f.iannazzo@policlinicoumberto1.it (F.I.); gadaleta.1861138@studenti.uniroma1.it (F.R.G.); edoardo.rosato@uniroma1.it (E.R.)

**Keywords:** renal artery stenosis, autonomic dysfunction, left ventricular mass index, uric acid, uric acid/creatinine ratio

## Abstract

Background: Serum uric acid and serum creatinine ratio (SUA/sCr) is strongly linked to increased cardiovascular risk. Atherosclerotic renal artery stenosis (ARAS) is a secondary cause of hypertension and is associated with ischemic nephropathy, congestive heart failure, accelerated cardiovascular disease, and autonomic dysfunction. The aim of this study was to investigate whether SUA levels and SUA/sCr could represent markers of autonomic dysfunction and increased left ventricular mass index (LVMI) in patients with ARAS. Methods: Patients diagnosed with ARAS were enrolled in the study. All patients underwent clinical evaluation, biochemical analysis, 24 h electrocardiogram (ECG), and Renal Doppler Ultrasound with renal resistive index parameters. Heart rate variability for global autonomic dysfunction was assessed through the analysis of a 24 h ECG to detect the standard deviation of normal-to-normal RR intervals (SDNN). Echocardiographic measurement of LVMI was performed. Results: A total of 27 patients (F = 16 (59%), median age 67 years (IQR 60–77)) diagnosed with ARAS were enrolled in the study. We found a statistically significant negative linear correlation between SUA/sCr and SDNN (r = −0.519, *p* < 0.01). We found a statistically significant positive linear correlation between SUA/sCr and LVMI (r = 0.413, *p* < 0.05). Median SDNN was significantly lower in patients with SUA ≥ 5.6 mg/dL than in patients with SUA < 5.6 mg/dL (94.2 (IQR 86.8–108.1) vs. 112.8 (IQR 108.9–114.7), *p* < 0.01). Median LVMI was significantly higher in patients with SUA ≥ 5.6 mg/dL compared to patients with SUA < 5.6 mg/dL (133 g/m^2^ (IQR 120–149) vs. 111 g/m^2^ (IQR 99–129), *p* < 0.05). Conclusion: In patients with ARAS, SUA/sCr is associated with autonomic dysfunction and LVMI in ARAS patients. The ratio and related cut-off value of SUA/sCr could represent a useful biomarker to evaluate cardiovascular risk in ARAS patients.

## 1. Introduction

Hypertension plays a significant role in the onset of cardiovascular and kidney diseases, contributing to higher rates of morbidity and mortality. Renal artery stenosis (RAS) is an important condition that affects approximately 1% to 6% of all hypertensive patients and can be found in up to 30% of those with atherosclerotic disease [1]. Atherosclerotic renal artery stenosis (ARAS), accounting for approximately 90% of RAS cases, is especially prevalent among individuals over the age of 50, with prevalence rates ranging from 10% to 15%. The percentage increases up to 60% in patients who have additional conditions, such as coronary atherosclerosis and renal damage [2,3]. Given its high prevalence, ARAS likely contributes significantly to the cardiovascular burden in these patients. In addition to hypertension, high levels of serum uric acid (SUA) have been found to negatively impact cardiovascular outcomes. Several observational studies have highlighted a strong association between high SUA levels and an increased risk of cardiovascular disease (CVD), including both clinical and subclinical manifestations [4]. Using survival receiver operating characteristic curve analysis, the optimal SUA cut-off for CV mortality was determined to be 5.6 mg/dL [4]. Elevated SUA levels are commonly found in patients with comorbidities like obesity, diabetes, and chronic kidney disease (CKD), conditions that are also prevalent in hypertensive patients with ARAS. Patients with ARAS and elevated SUA levels are associated with higher blood pressure and left ventricular hypertrophy (LVH), as indicated by an increased left ventricular mass index (LVMI) [5]. This phenomenon may be attributed to several factors, including the activation of the renin-angiotensin system, which can lead to increased blood pressure; a reduction in vascular nitric oxide production, which is essential for vasodilation; or the activation of sodium channels in the distal nephron, which can contribute to sodium retention and further elevate blood pressure [5]. Additionally, ARAS often leads to diastolic dysfunction, which increases the risk of heart failure with preserved ejection fraction (HFpEF). Diastolic dysfunction, characterized by impaired LV relaxation and increased myocardial stiffness, is associated with higher mortality rates [6]. Hypertensive patients with ARAS exhibit a higher prevalence of LVH and diastolic dysfunction, highlighting the importance of careful monitoring and management of this group to prevent further cardiovascular complications. Similarly, SUA levels are independently associated with increased left ventricular mass and the development of LVH, making SUA a key factor in predicting cardiovascular outcomes in hypertensive patients. The combination of hyperuricemia and LVH further increases the risk of cardiovascular death, underscoring the need for a more integrated approach to managing these patients [7].

Furthermore, autonomic dysfunction is a well-established risk factor for CVD and mortality. It has been associated with an increased risk of atherosclerosis [8]. Although the exact mechanisms linking autonomic imbalance to atherosclerosis remain unclear, recent studies suggest a connection between autonomic function and inflammation in CVD patients [8]. In particular, an inverse relationship has been observed between autonomic activity, measured by heart rate variability (HRV), and plasma levels of inflammatory markers [8]. Recently, in our group, we found that global autonomic activity was significantly lower in ARAS patients than healthy controls [9].

Given the high cardiovascular risk in ARAS patients, the aim of this study was to investigate whether SUA levels and SUA/sCr could represent markers of autonomic dysfunction and increased LVMI in patients with ARAS.

## 2. Materials and Methods

We performed a cross-sectional study based on patients who were eligible for inclusion if they had ARAS (diagnosed with Doppler ultrasound), whose blood pressure was controlled with medication, and whose condition was not suitable for percutaneous revascularization.

All patients underwent a clinical evaluation, biochemical analysis, electrocardiography (ECG) 24 h, and Renal Doppler Ultrasound with renal resistive index (RRI) evaluation. The RRI was calculated by dividing the difference between peak systolic velocities (PSV) and end-diastolic velocities (EDV) by the PSV:RRI = [(PSV − EDV)/PSV].

Patients with heart diseases, arrhythmia, and conditions that could interfere with autonomic activity, such as diabetes mellitus, were excluded. Renal fibromuscular dysplasia was also excluded due to its different pathogenesis compared to ARAS. Patients did not take anti-arrhythmic and QTc prolonging drugs. Patients receiving β-blockers therapy underwent HRV examination one week after discontinuation. The subjects’ written consent was obtained, and the study was conducted according to the Declaration of Helsinki. The study was approved by the Ethics Committee of Sapienza University of Rome (IRB n° 0304).

### 2.1. Data Collection

SUA levels were collected from all patients. Systolic blood pressure (SBP) and diastolic blood pressure (DBP) were measured twice, in a quiet room, after 5 min of resting and with the participant in a sitting position. The second determination was used for the analysis. Renal function was evaluated through estimation of the glomerular filtration rate (GFR), according to the Chronic Kidney Disease Epidemiology Collaboration equation (CKD-EPI) [10].

### 2.2. Heart Rate Variability and QTc Interval

Autonomic nervous system activity was evaluated by HRV analysis during a 24 h ECG recording following the recommendations of the Task Force of the European Society of Cardiology and the North American Society of Pacing and Electrophysiology [11]. The patients underwent a 24 h ambulatory, 3-channel Holter ECG recording (Lifecard CF; Spacelabs Healthcare, Snoqualmie, WA, USA). At 10 min intervals, all the participants were studied with ECG recordings. Artificial and arrhythmic data were not considered. In the time domain, we evaluated the standard deviation of normal-to-normal RR intervals (SDNN) (ms), which represents global autonomic activity, and the square root of the mean of the sum of the squares of differences between adjacent NN intervals (rMSSD), which represents a marker of global parasympathetic system. QTc interval, assessed by 24-h Holter ECG recording, was defined as prolonged when >440 ms [12].

### 2.3. Left Ventricular Mass and Left Ventricular Mass Index

To determine LVM (in grams), patients with ARAS underwent bidimensional M-mode echocardiography using the Devereux regression formula. According to the literature, normal LVM values were considered ≤115 g in men and ≤95 g in women [13]. The absolute value of LVM was then normalized by the patient’s body surface area (LVM index (LVMI), calculated as grams divided by meters squared).

### 2.4. Statistical Analysis

SPSS version 26.0 software was used for statistical analysis. After evaluation of normality, continuous variables were expressed as median and interquartile range (IQR), since data were not normally distributed. Student’s or Mann–Whitney’s *t*-test was used to evaluate differences between groups. Bonferroni’s corrections were applied in case of multiple comparisons. The chi-square or Fisher exact test was used to evaluate differences between categorical variables. The Pearson or Spearman correlation test was used for bivariate correlations. Receiver operating characteristic (ROC) curve analysis was performed to analyze the prognostic accuracy of a cut-off of SUA/sCr ≥ 4.05 for RRI and of a cut-off of SUA ≥ 5.6 mg/dL for SDNN and LVMI. *p*-value < 0.05 was considered significant.

## 3. Results

A total of 27 patients (F = 16 (59%), median age 67 years (IQR 60–77)) were enrolled in this study. Comorbidities of patients enrolled were: smoking (33.3%), acute myocardial infarction (11.1%), CKD (29.6%), and dyslipidemia (66.7). Median SBP and DBP were 130 mmHg (IQR 125–140) and 80 mmHg (IQR 75–80), respectively. Median sCr was 1.05 mg/dL (IQR 0.8–1.3) with a median eGFR of 67.5 mL/min (IQR 53.7–89.2), according to the CKD-EPI equation. Median SUA was 5.3 mg/dL (IQR 4.6–6.3), and 9 (33.3%) patients had SUA ≥ 5.6 mg/dL. Median SUA/sCr was 4.92 (IQR 4–7.06), and 19 (70.4%) patients had SUA/sCr ≥ 4.05. Median RRI was 0.7 (IQR 0.63–0.72), and 13 (48.1%) patients had RRI ≥ 0.7. Median SDNN was 111.2 (IQR 106.3–114.2) and median LVMI was 120 g/m^2^ (IQR 100–133). Demographic and clinical characteristics of patients enrolled are shown in Table 1.

**Figure 1:**
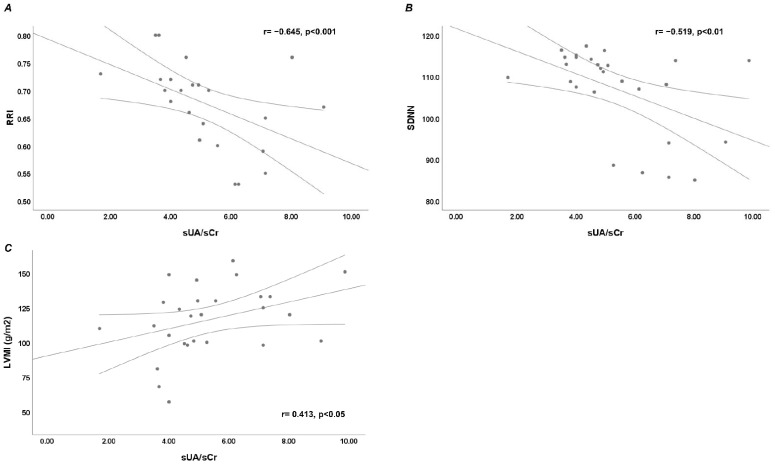
Bivariate linear correlation between serum uric acid (sUA)/serum creatinine (sCr) ratio and parameters of renal and cardiac involvement. (**A**) Bivariate linear correlation between sUA/sCr and renal resistive index (RRI); (**B**) Bivariate linear correlation between sUA/sCr and Standard Deviation Normal to Normal (SDNN); (**C**) Bivariate linear correlation between sUA/sCr and left ventricular mass index (LVMI).

We found a statistically significant negative linear correlation between SUA/sCr and RRI (r = −0.645, *p* < 0.001) (Figure 1A). Moreover, median RRI was significantly lower in patients with SUA/sCr ≥ 4.05 compared to patients with SUA/sCr < 4.05 (0.66 (IQR 0.6–0.7) vs. 0.72 (IQR 0.72–0.80), *p* < 0.01). We found a statistically significant negative linear correlation between SUA/sCr and SDNN (r = −0.519, *p* < 0.01) (Figure 1B). We found a statistically significant positive linear correlation between SUA/sCr and LVMI (r = 0.413, *p* < 0.05) (Figure 1C).

Median SDNN was significantly lower in patients with SUA ≥ 5.6 mg/dL than in patients with SUA < 5.6 mg/dL (94.2 (IQR 86.8–108.1) vs. 112.8 (IQR 108.9–114.7), *p* < 0.01). Median LVMI was significantly higher in patients with SUA ≥ 5.6 mg/dL compared to patients with SUA < 5.6 mg/dL (133 g/m^2^ (IQR 120–149) vs. 111 g/m^2^ (IQR 99–129), *p* < 0.05). No significant correlation was found between rMSSD and the variables considered (*p* > 0.05).

**Figure 2:**
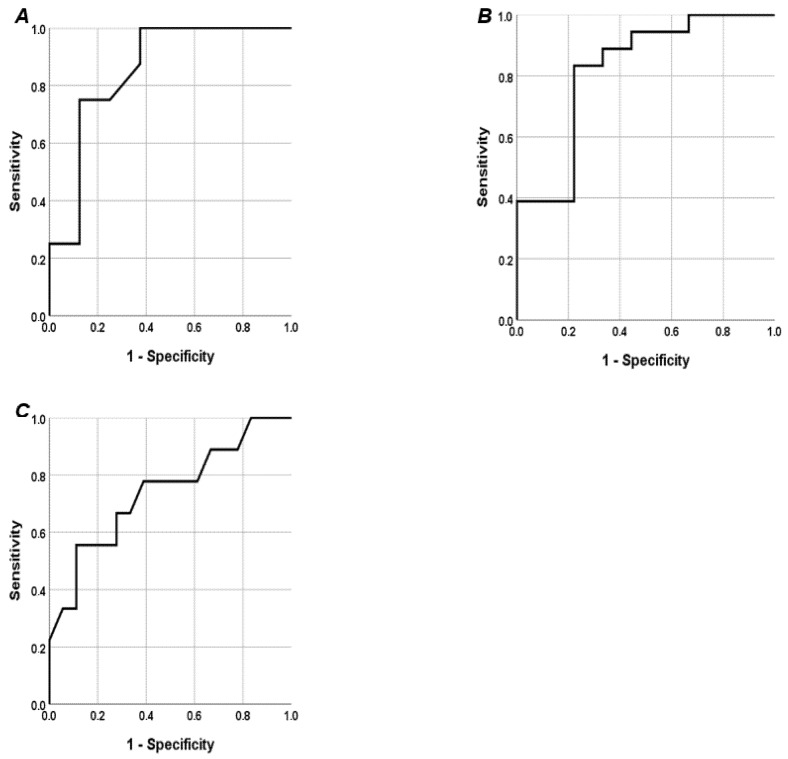
Receiver operating characteristic (ROC) curve. (**A**) ROC curve performed to analyze the prognostic accuracy of a cut-off of SUA/sCr ≥ 4.05 for RRI; (**B**) ROC curve performed to analyze the prognostic accuracy of SUA ≥ 5.6 mg/dL for Standard Deviation Normal to Normal (SDNN); (**C**) ROC curve performed to analyze the prognostic accuracy of SUA ≥ 5.6 mg/dL for left ventricular mass index (LVMI).

The ROC curves showed a good prognostic accuracy of a cut-off of SUA/sCr ≥ 4.05 for RRI (AUC 0.852 (95% CI 0.698–1), *p* < 0.01) (Figure 2A). The ROC curves showed a good prognostic accuracy of a cut-off of SUA ≥ 5.6 mg/dL for SDNN (AUC 0.821 (95% CI 0.642–1), *p* < 0.01) (Figure 2B). The ROC curves showed a good prognostic accuracy of a cut-off of SUA ≥ 5.6 mg/dL for LVMI (AUC 0.741 (95% CI 0.532–0.950), *p* < 0.05) (Figure 2C).

## 4. Discussion

The main finding of our study is that SUA/sCr is associated both with autonomic dysfunction and LVMI in patients with ARAS. Hyperuricemia is connected to high blood pressure, diabetes, dyslipidemia, obesity, CKD, and coronary artery disease, and it is closely related to increased CV risk [14]. Recently, in the multicenter study of the Working Group on Uric Acid and Cardiovascular Risk of the Italian Society of Hypertension, the authors found that SUA/sCr was a predictor of cardiovascular events [15]. SUA is closely associated with kidney function, making the SUA/SCr ratio a more dependable indicator, particularly when kidney damage is present, as a marker of cardiovascular risk and disease [16]. Different mechanisms have been identified in the kidney and vascular damage caused by SUA [17]. The role of hyperuricemia in hypertensive nephrosclerosis is well documented [18]. Also, Yu et al. found that SUA levels and tubular atrophy and interstitial fibrosis are independent risk factors of glomerular ischemic lesions in patients with primary membranous glomerulonephritis (GN) [19].

Recently, in a retrospective observational study, our group found the correlation between SUA level and SUA/SCr in the development of chronic and vascular lesions (CVL) in patients with primary GN. Hyperuricemia is often associated with CKD, representing a risk factor for the progression of renal disease. Uric acid, a byproduct of purine breakdown, is primarily eliminated by the kidneys, leading to vascular alterations such as damage to the arterioles preceding the glomeruli, characterized by hyalin deposition and thickening of the vessel walls [20].

In the Kailuan Study, patients with CKD and higher SUA levels were associated with a higher risk of myocardial infarction, heart failure, and all-cause mortality [21]. Autonomic dysfunction can negatively impact cardiac electrophysiology in CKD patients, leading to increased sympathetic activity alongside a reduction in parasympathetic tone [22].

HRV, a way to indirectly assess the interplay between the sympathetic and parasympathetic nervous systems at the heart’s sinoatrial node, provides insight into autonomic balance [23].

Previously, an elevated SUA level has been identified as an independent predictor of hypertension. Its strong correlation with sympathetic parameters, particularly in prehypertensive individuals, suggests a potential role for SUA in sympathetic autonomic dysregulation [24]. This connection is further supported by evidence linking inflammation, SUA levels, and autonomic dysfunction to different stages of hypertension [25]. A large-scale study in a Chinese population by Liao et al. further explored this relationship, revealing interactive effects of SUA and hypertension on cardiovascular autonomic neuropathy (CAN). Their findings indicate that hypertensive patients with high uric acid are more prone to the progressive impact of CAN [26].

In our study, a statistically significant negative linear correlation between SUA/sCr and SDNN was found. Furthermore, median SDNN was significantly lower in patients with SUA ≥ 5.6 mg/dL [4]. Few studies in the literature have addressed the role of autonomic dysfunction in ARAS [9].

The pathogenesis of ARAS is driven by the activation of pro-inflammatory and pro-fibrotic mechanisms. A significant reduction in blood flow initiates a cascade of hormonal responses, such as the renin angiotensin aldosterone system (RAAS). Importantly, this activation significantly increases sympathetic nerve activity, contributing to the rise in arterial pressure alongside arterial changes, vasoconstriction, sodium retention, and inflammation [27].

Findings from animal studies suggest that high UA is a potent contributor to high blood pressure through a complex interplay of biological processes. Elevated UA appears to initiate a cascade involving the activation of RAAS. This activation is coupled with increased oxidative stress, the development of vascular insulin resistance, and a reduction in the crucial vasodilator, endothelial nitric oxide, ultimately leading to elevated blood pressure [28].

In this scenario, it is not surprising that SUA/sCr correlates with SDNN in ARAS patients.

A significant negative linear correlation between SUA/sCr and RRI was found. In a previous study in patients with ARAS, we demonstrated that at baseline, high SUA levels, elevated RRI, and proteinuria were predictive factors for worsened renal function following revascularization [29]. It is well known that hyperuricemia is the underlying cause of arteriolopathy in the preglomerular vessels, which in turn compromises renal autoregulation. The resulting lumen obliteration can produce severe renal hypoperfusion, ultimately contributing to tubulointerstitial inflammation, fibrosis, and arterial hypertension.

On the other hand, CKD impairs renal UA handling [30]. In the Concentration of serum uric acid in patients with Renal Artery Stenosis and Hypertension prEdict Future nephropathy and death, “C-RASHEF” study, the authors found that SUA has a differential impact on mortality and new or worsening nephropathy prediction in patients with and without RAS [31]. The causal link between hyperuricemia and oxidative stress generation, mainly mediated by NADPH activation, is well-established. Importantly, cellular uric acid uptake has been identified as a critical step driving the development and progression of organ damage.

The relationship between SUA and CV events has been well documented [32]. Also in the present study, we found a statistically significant positive linear correlation between SUA/sCr and LVMI, and median LVMI was significantly higher in patients with SUA ≥ 5.6 mg/dL. Several important studies link SUA to increased LV mass [7,33,34,35]. This finding, initially in a limited group, was validated in a larger population [35]. In our study, the ROC curves showed a good prognostic accuracy of a cut-off of SUA ≥ 5.6 mg/dL for LVMI.

The relationship between SUA levels and CV disease has been a subject of study for many years. With the rising prevalence of high UA levels due to unhealthy lifestyles, this connection is expected to gain greater significance, although the exact way UA contributes to heart disease is still not fully understood. While a connection is evident, a definitive causal relationship is not yet established. Ongoing research is crucial to identify a clear pathophysiological mechanism, which could reveal a potential target for therapeutic intervention. Given that ARAS can lead to various adverse complications, including secondary hypertension, ischemic nephropathy, left ventricular dysfunction, pulmonary edema, and cerebrocardiovascular events, it is fundamental to identify markers, especially because not all patients derive clinical benefit from renal angioplasty. Indeed, current guidelines need to define more precisely the factors associated with potential benefits for these patients.

The small sample size is the primary limitation of this study. Future studies ought to include frequency domain parameters in addition to the time domain in order to provide a more comprehensive assessment of HRV.

## 5. Conclusions

In conclusion, this study demonstrated that the SUA/sCr is independently associated with both autonomic dysfunction and LVMI in patients with ARAS. The ratio, and its related cut-off values, represents an independent and potentially useful biomarker for assessing cardiovascular risk in this patient population, as the association between SUA/sCr and cardiovascular events may be explained in part by its direct correlation with both autonomic function and left ventricular mass. Further studies involving a larger cohort of ARAS patients should confirm these findings.

## Figures and Tables

**Table 1:** Demographic and clinical characteristics of 27 patients enrolled. Results are reported as median and interquartile range (IQR) or absolute frequency and percentage (%).

Age, years	67 (60–77)
Female/Male	16 (59)/11 (41)
BMI, kg/m^2^	24.3 (23.2–27.8)
SBP, mmHg	130 (125–140)
DBP, mmHg	80 (75–80)
eGFR (CKD-EPI), mL/min	67.5 (53.7–89.2)
sCr, mg/dL	1.05 (0.8–1.3)
SUA, mg/dL	5.3 (4.6–6.3)
SUA ≥ 5.6, mg/dL	9 (33.3)
SUA/sCr	4.92 (4–7.06)
SUA/sCr ≥ 4.05	19 (70.4)
Na	140 (139–143)
K	4.5 (4.27–4.9)
Ca	9.67 (9.4–10)
P	3.75 (3.4–4)
RRI	0.7 (0.63–0.72)
RRI ≥ 0.7	15 (55.5)
SDNN	111.2 (106.3–114.2)
HR mean	78.6 (73.9–83.6)
HR min	73.5 (70–79)
HR max	113 (104–118)
QTc mean	436 (418–458)
LVMI, g/m^2^	120 (100–133)

Abbreviations: BMI: body mass index; SBP: systolic blood pressure; DBP: diastolic blood pressure; eGFR: estimated Glomerular Filtration Rate; CKD-EPI: Chronic Kidney Disease Epidemiology Collaboration; sCr: serum creatinine; SUA: serum uric acid; RRI: renal resistive index; SDNN: standard deviation of normal-to-normal RR intervals; HR: heart rate; LVMI: left ventricular mass index.

## Data Availability

Data were anonymized for this study. All data are available upon request to the corresponding author.

## References

[1] Li Y., Chen Z., Lan R., Ran T., He J., Li J., Shi Q., Mao M., Zuo Z. (2024). Atherosclerotic renal artery stenosis, mediating biomarkers, and risk of cardiac among individuals with hypertension: A real-world study. Int. J. Cardiol. Heart Vasc..

[2] de Mast Q., Beutler J.J. (2009). The prevalence of atherosclerotic renal artery stenosis in risk groups: A systematic literature review. J. Hypertens..

[3] Safian R.D., Textor S.C. (2001). Renal-artery stenosis. N. Engl. J. Med..

[4] Virdis A., Masi S., Casiglia E., Tikhonoff V., Cicero A.F.G., Ungar A., Rivasi G., Salvetti M., Barbagallo C.M., Bombelli M. (2020). Identification of the Uric Acid Thresholds Predicting an Increased Total and Cardiovascular Mortality over 20 Years. Hypertension.

[5] Chen X.J., Eirin A., Kane G.C., Misra S., Textor S.C., Lerman A., Lerman L.O. (2019). Impact of Serum Uric Acid Levels on Outcomes following Renal Artery Revascularization in Patients with Renovascular Disease. Int. J. Hypertens..

[6] Deswal A. (2005). Diastolic dysfunction and diastolic heart failure: Mechanisms and epidemiology. Curr. Cardiol. Rep..

[7] Kjeldsen S.E., Mariampillai J.E., Høieggen A. (2023). Uric acid and left ventricular mass in prediction of cardiovascular risk-New insight from the URRAH study. Eur. J. Intern. Med..

[8] Ulleryd M.A., Prahl U., Börsbo J., Schmidt C., Nilsson S., Bergström G., Johansson M.E. (2017). The association between autonomic dysfunction, inflammation and atherosclerosis in men under investigation for carotid plaques. PLoS ONE.

[9] Gigante A., Zingaretti V., Proietti M., Rosato E., Cianci R. (2018). Autonomic dysfunction and cardiovascular risk in patients with atherosclerotic renal artery stenosis: A pilot study. Eur. J. Intern. Med..

[10] Levey A.S., Stevens L.A., Schmid C.H., Zhang Y.L., Castro A.F., Feldman H.I., Kusek J.W., Eggers P., Van Lente F., Greene T. (2009). A new equation to estimate glomerular filtration rate. Ann. Intern. Med..

[11] Malik M. (1996). Heart rate variability: Standards of measurement, physiological interpretation, and clinical use. Task Force of the European Society of Cardiology and the North American Society of Pacing and Electrophysiology. Circulation.

[12] Moss A.J. (1993). Measurement of the QT interval and the risk associated with QTc in interval prolongation: A review. Am. J. Cardiol..

[13] Lang R.M., Bierig M., Devereux R.B., Flachskampf F.A., Foster E., Pellikka P.A., Picard M.H., Roman M.J., Seward J., Shanewise J. (2006). Recommendations for chamber quantification. Eur. J. Echocardiogr..

[14] Kuwabara M., Hisatome I., Ae R., Kosami K., Aoki Y., Andres-Hernando A., Kanbay M., Lanaspa M.A. (2025). Hyperuricemia, A new cardiovascular risk. Nutr. Metab. Cardiovasc. Dis..

[15] Casiglia E., Tikhonoff V., Virdis A., Grassi G., Angeli F., Barbagallo C.M., Bombelli M., Cicero A.F.G., Cirillo M., Cirillo P. (2023). Serum uric acid/serum creatinine ratio as a predictor of cardiovascular events. Detection of prognostic cardiovascular cut-off values. J. Hypertens..

[16] D’Elia L., Masulli M., Cirillo P., Virdis A., Casiglia E., Tikhonoff V., Angeli F., Barbagallo C.M., Bombelli M., Cappelli F. (2024). Serum uric acid/serum creatinine ratio and cardiovascular mortality in diabetic individuals-the uric acid right for heart health (URRAH) project. Metabolites.

[17] Russo E., Bertolotto M., Zanetti V., Picciotto D., Esposito P., Carbone F., Montecucco F., Pontremoli R., Garibotto G., Viazzi F. (2023). Role of uric acid in vascular remodeling: Cytoskeleton changes and migration in VSMCs. Int. J. Mol. Sci..

[18] Sofue T. (2023). Hyperuricemia: The third key player for nephrosclerosis with ischemia. Hypertens. Res..

[19] Yu Y., Zheng J., Li J., Li X., Liu Z., Yang R., Hong H., Zhang J. (2024). Serum uric acid level is associated with glomerular ischemic lesions in patients with primary membranous nephropathy: An analytical, cross-sectional study. Sci. Rep..

[20] Gigante A., Pellicano C., Gallicchio C., Melena M., Fiorino M., Rosato E., Giannakakis K., Ascione A., Muscaritoli M., Cianci R. (2025). Serum Uric Acid/Serum Creatinine Ratio and Chronic Vascular Lesions on Renal Biopsy: A Retrospective Observational Study. High. Blood Press. Cardiovasc. Prev..

[21] Li N., Cui L., Shu R., Song H., Wang J., Chen S., Han Y., Yu P., Yuan W., Wang J. (2024). Associations of uric acid with the risk of cardiovascular disease and all-cause mortality among individuals with chronic kidney disease: The Kailuan Study. Eur. J. Prev. Cardiol..

[22] Lai S., Bagordo D., Perrotta A.M., Gigante A., Gasperini M.L., Muscaritoli M., Mazzaferro S., Cianci R. (2020). Autonomic dysfunction in kidney diseases. Eur. Rev. Med. Pharmacol. Sci..

[23] Shaffer F., Ginsberg J.P. (2017). An Overview of Heart Rate Variability Metrics and Norms. Front. Public Health.

[24] Kunikullaya K.U., Purushottam N., Prakash V., Mohan S., Chinnaswamy R. (2015). Correlation of serum uric acid with heart rate variability in hypertension. Hipertens. Riesgo Vasc..

[25] Erden M., Kocaman S.A., Poyraz F., Topal S., Sahinarslan A., Boyacı B., Cengel A., Yalçın M.R. (2011). Incremental effects of serum uric acid levels, autonomic dysfunction, and low-grade inflammation on nocturnal blood pressure in untreated hypertensive patients and normotensive individuals. Turk. Kardiyol. Dern. Ars..

[26] Liao X.P., Zhu H.W., Zeng F., Tang Z.H. (2015). The association and interaction analysis of hypertension and uric acid on cardiovascular autonomic neuropathy. J. Endocrinol. Investig..

[27] Hicks C.W., Clark T.W.I., Cooper C.J., de Bhailís Á.M., De Carlo M., Green D., Małyszko J., Miglinas M., Textor S.C., Herzog C.A. (2022). Atherosclerotic Renovascular Disease: A KDIGO (Kidney Disease: Improving Global Outcomes) Controversies Conference. Am. J. Kidney Dis..

[28] Tao M., Pi X., Ma X., Shi Y., Zhang Y., Gu H., Chi Y., Zhuang S., Liu N. (2019). Relationship between serum uric acid and clustering of cardiovascular disease risk factors and renal disorders among Shanghai population: A multicentre and cross-sectional study. BMJ Open.

[29] Cianci R., Martina P., Gigante A., Di Donato D., Polidori L., Presta P., Labbadia R., Amoroso D., Zaccaria A., Barbano B. (2013). Predictor factors for renal outcome in renal artery stenosis. Eur. Rev. Med. Pharmacol. Sci..

[30] Russo E., Viazzi F., Pontremoli R., Barbagallo C.M., Bombelli M., Casiglia E., Cicero A.F.G., Cirillo M., Cirillo P., Desideri G. (2022). Association of uric acid with kidney function and albuminuria: The Uric Acid Right for heArt Health (URRAH) Project. J. Nephrol..

[31] Zheng B. (2023). Concentration of serum uric acid in patients with Renal Artery Stenosis and Hypertension prEdict Future nephropathy and death: C-RASHEF study. J. Clin. Hypertens..

[32] Muiesan M.L., Agabiti Rosei C., Paini A., Casiglia E., Cirillo M., Grassi G., Iaccarino G., Mallamaci F., Maloberti A., Mazza A. (2023). Serum uric acid and left ventricular mass index independently predict cardiovascular mortality: The uric acid right for heart health (URRAH) project. Eur. J. Intern. Med..

[33] Viazzi F., Parodi D., Leoncini G., Parodi A., Falqui V., Ratto E., Vettoretti S., Bezante G.P., Del Sette M., Deferrari G. (2005). Serum uric acid and target organ damage in primary hypertension. Hypertension.

[34] Catena C., Colussi G., Capobianco F., Brosolo G., Sechi L.A. (2014). Uricaemia and left ventricular mass in hypertensive patients. Eur. J. Clin. Investig..

[35] Visco V., Pascale A.V., Virtuoso N., Mongiello F., Cinque F., Gioia R., Finelli R., Mazzeo P., Manzi M.V., Morisco C. (2020). Serum uric acid and left ventricular mass in essential hypertension. Front. Cardiovasc. Med..

